# Dialectical Behavior Therapy and Cognitive Behavior Therapy in Individuals With Binge‐Eating Disorder: What Works for Whom?

**DOI:** 10.1111/eat.70000

**Published:** 2025-11-21

**Authors:** Mirjam W. Lammers, Maartje S. Vroling, Ross D. Crosby, Suzanne H. W. Mares, Giel J. M. Hutschemaekers, Tatjana Van Strien

**Affiliations:** ^1^ Amarum, Expertise Centre for Eating Disorders, GGNet Network for Mental Health Care Zutphen the Netherlands; ^2^ Radboud University, Behavioural Science Institute Nijmegen the Netherlands; ^3^ Sanford Center for Biobehavioral Research Fargo North Dakota USA; ^4^ University of North Dakota School of Medicine and Health Sciences Fargo North Dakota USA; ^5^ Department of Clinical Psychology Utrecht University Utrecht the Netherlands

**Keywords:** binge‐eating disorder, cognitive behavior therapy, dialectical behavior therapy, moderators, predictors

## Abstract

**Objective:**

To evaluate moderators and predictors of response to cognitive behavioral therapy (CBT) and dialectical behavior therapy (DBT) for binge‐eating disorder (BED).

**Method:**

Moderators/predictors of treatment outcome, central to CBT and to DBT treatment, were chosen from an aggregated dataset of two clinical outcome studies with non‐random allocation to treatment groups (*N* = 203). Both studies compared DBT‐BED (*n* = 71) to a more intensive outpatient CBT program (CBT+, *n* = 132) in individuals with BED. Generalized linear models examined moderators and predictors of objective binge‐eating (OBE) frequency at end of treatment (EOT) and six‐month follow up (FU).

**Results:**

Baseline shape/weight overvaluation, shape concerns and low self‐esteem significantly predicted and moderated reductions in OBE frequency at EOT whereas difficulty in identifying feelings predicted and moderated outcome at FU. Emotional eating predicted outcome at FU (medium effect) and moderated outcome at EOT (large effect). Depression levels predicted, but not moderated, treatment outcome at both EOT and FU. For shape/weight overvaluation, shape concerns and low self‐esteem, low levels were related to fewer reductions in outcome in DBT‐BED than in CBT+. Individuals with the highest levels of emotional eating and difficulty identifying feelings showed more decrease in OBE episodes with DBT‐BED than with CBT+ at EOT and FU respectively.

**Discussion:**

Findings suggest that BED treatment outcomes could be enhanced by matching individuals with certain symptom presentations to treatment overall, and to DBT‐BED or CBT+ specifically. DBT‐BED may be a promising alternative to CBT+ for those with more severe psychopathology.

**Summary**

- This study suggests that BED treatment outcome could be enhanced by matching individuals with certain symptom presentations to treatment overall and to DBT‐BED or CBT+ specifically.
- Particularly levels of emotional eating, difficulties identifying feelings, shape/weight overvaluation, shape concerns and low self‐esteem may inform treatment plans.
- In general, DBT‐BED may be a promising alternative to CBT+ for those BED patients with more severe psychopathology.

## 1 Introduction

Individuals with binge‐eating disorder (BED) suffer from recurrent, psychologically distressing, brief episodes of uncontrollable overeating (American Psychiatric Association 2013). BED has been linked to psychiatric and medical comorbidities as well as impaired social functioning (Brownley et al. 2016; Grucza et al. 2007; Welch et al. 2016). Even with the most effective treatments, cognitive behavior therapy (CBT) and interpersonal psychotherapy (IPT), about 50% of patients remain symptomatic (Linardon 2018). To improve abstinence rates, new BED‐treatments were developed, such as dialectical behavior therapy (DBT) (Ben‐Porath et al. 2020; Linardon, Fairburn, et al. 2017). DBT was originally developed to treat ongoing self‐harm and suicidal behaviors in patients with borderline personality disorder (Linehan 1993). The adapted form for BED (DBT‐BED) teaches emotion regulation skills in order to replace binge eating as a way of coping with negative affect (Safer et al. 2009; Telch et al. 2001). The therapy leads to large pre‐post symptom improvements (e.g., Blood et al. 2020) and is consistently more efficacious than waitlist controls (Masson et al. 2013; Rahmani et al. 2018; Telch et al. 2001). Although CBT may be more effective than DBT‐BED in the short term (Lammers et al. 2020, 2021), these differences disappear in the longer run (Chen et al. 2017; Lammers et al. 2020, 2022). Nevertheless, as each treatment theoretically acts through different mechanisms, some patients may profit more from one treatment than the other. The identification of baseline variables that moderate responses to treatment could help clinicians make well‐informed, effective decisions about what works for whom (Kraemer et al. 2002).

To date, most of the research on moderators of treatment outcomes for BED concerns manualized CBT compared to other treatments including IPT, CBT‐guided self‐help (CBT‐gsh), behavioral weight loss and medication (Grilo, Gueorguieva, and Pittman 2021; Linardon, de la Piedad Garcia, and Brennan 2017). Results are poor, as most assumed moderators (e.g., demographics, global eating disorder psychopathology and depression scores) do not show any differential effect. Exceptions are age of binge‐eating onset, weight concern and overvaluation of weight and shape which differentially predicted outcome in some studies (Grilo, Thompson‐Brenner, et al. 2021; Linardon, de la Piedad Garcia, and Brennan 2017). Only two studies have evaluated moderators of response to emotion‐focused interventions for BED relative to other treatments. In the first study (Robinson and Safer 2012), an earlier onset of overweight and dieting (< 15 years old) as well as avoidant personality disorder signaled poorer outcome when treated with a non‐specific supportive psychotherapy versus DBT‐BED. In the second study, low shape/weight overvaluation, greater pretreatment self‐control and greater baseline actual‐ideal self‐discrepancy predicted greater reductions in OBE frequency at end of treatment (EOT) in integrative cognitive affective therapy for BED (ICAT‐BED) than in CBT‐gsh (Anderson et al. 2020). So far, no study has tested moderators of response comparing CBT and DBT‐BED. The present study aims to fill this gap.

### 1.1 The Current Study

**FIGURE 1:**
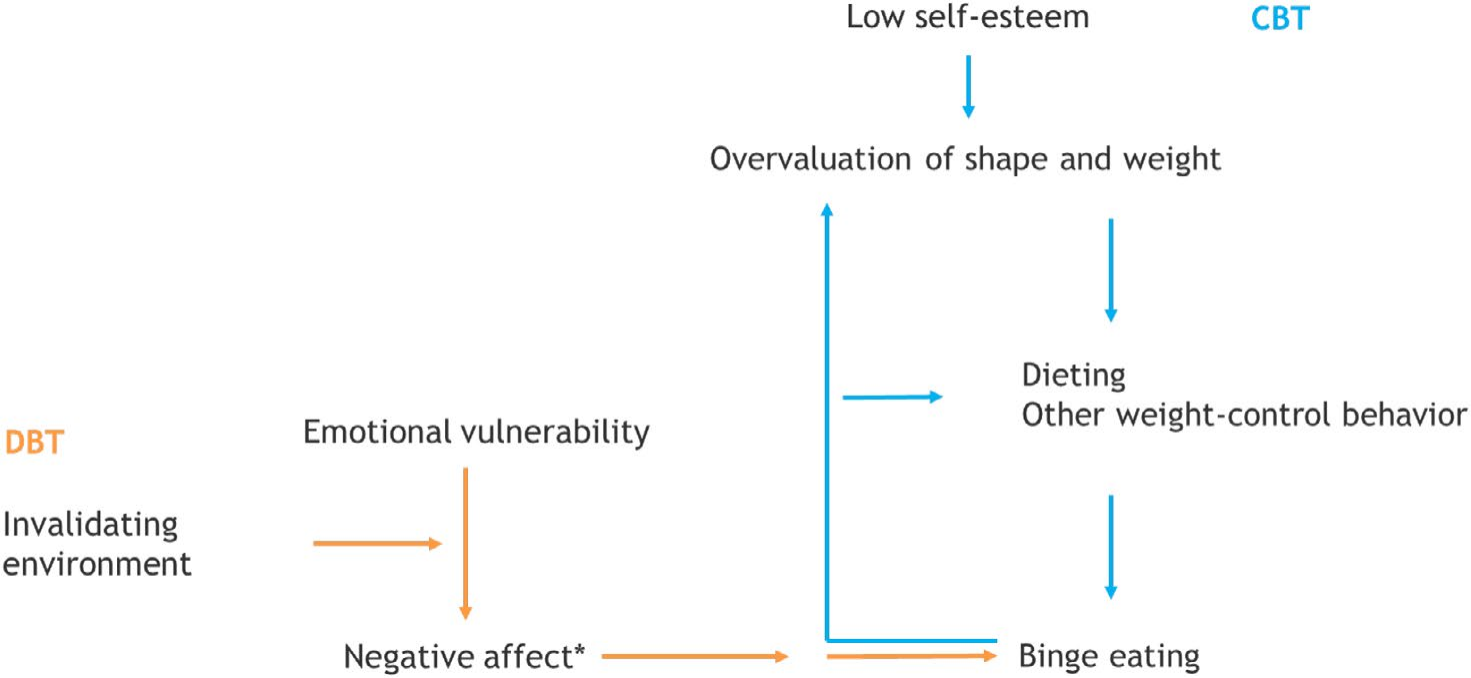
CBT and DBT‐treatment models of binge eating. *: Although, in CBT, binge eating may also be maintained by negative affect, the main focus in CBT is on the link between overvaluation, dieting and binge eating.

This study explores whether baseline characteristics differentially predict treatment outcome for DBT‐BED and CBT at EOT and at six‐month follow‐up (FU) in individuals with (subthreshold) BED. Also, predictor main effects are tested as results inform us on who profits more or less from treatment, irrespective of treatment type (Kraemer et al. 2002). Available data from a quasi‐randomized controlled trial (Lammers et al. 2020) and an effectiveness study (Lammers et al. 2022) will be used. Both studies compared group DBT‐BED to a more intensive outpatient CBT group‐program (CBT+), based on CBT for binge eating (Fairburn et al. 1993). In the effectiveness study, both therapist variables (e.g., ideas about which treatment fits which case conceptualization best), availability and, ultimately, patient preference (e.g., for a certain treatment day, treatment intensity or treatment content) played a part in the decision whether to start in CBT+ or DBT‐BED. The CBT‐model considers binge eating to be maintained by dieting and possible other non‐compensatory weight‐control behavior, which follows from the overvaluation of body shape and/or body weight. This, in turn, is maintained by low self‐esteem. The aim is to stop binge eating by implementing a balanced eating pattern and by diminishing the influence of body shape and weight on self‐esteem. The DBT‐model assumes that binge eating serves as a way to regulate negative affect via negative reinforcement (Safer et al. 2009; Telch et al. 2001). DBT‐BED focuses on replacing binge eating by adaptive emotion regulation skills. In the original outcome study (Lammers et al. 2020), we decided to stay close to clinical practice in order to optimize generalizability and thus did not control the two treatments for content. Therefore, some conceptual overlap may have occurred as in CBT+ some emotion regulation strategies are introduced as one of the ways to deal with triggers for binge eating, and DBT‐BED incorporates education on a balanced eating pattern and regular physical exercise to diminish the sensitivity for negative emotions. Nevertheless, we hypothesize that patients with more distinct emotion regulation problems at baseline demonstrate greater symptom improvements in DBT‐BED, whereas those with greater difficulties in areas related to dieting and overvaluation of shape or weight, fare better with CBT. Because the CBT‐model (Fairburn et al. 2003) posits a more indirect link between self‐esteem and overeating, we tentatively hypothesize that for those with lower self‐esteem, CBT leads to better outcome. Figure 1 shows CBT and DBT‐treatment models of binge eating.

## 2 Method

### 2.1 Participants

Participants were referred to a Dutch community eating disorder service. Individuals, aged 16 years or older, who met DSM‐5 criteria for BED (APA 2013) or subthreshold BED (BED of low frequency and those with subjective binge‐eating episodes) were eligible. Exclusion criteria were: concurrent treatment for BED or weight problems (but including those who had undergone bariatric surgery), occasional purging behavior, comorbid psychiatric conditions that require immediate attention (e.g., acute psychosis and suicidality), medical conditions that preclude treatment of the eating disorder and conditions that warrant individual rather than group treatment (e.g., intellectual disability). Following an initial telephone screening, a clinical interview resulting in a case formulation and a DSM‐5 classification was conducted to verify eligibility. Participants were enrolled in either a quasi‐randomized controlled trial (*N* = 74: Lammers et al. 2020), or a naturalistic effectiveness study (*N* = 175: Lammers et al. 2022). Both trials compared intensive outpatient CBT (CBT+) and DBT‐BED. The pooled data set included 249 participants, 166 that received CBT+ and 83 that received DBT‐BED. All participants completed self‐report questionnaires assessing variables of interest at baseline. EOT assessments were obtained for 203 (81.5%) participants, including 132 (79.5%) in CBT+ and 71 (85.5%) in DBT‐BED. FU assessments were obtained for 142 (57.0%) participants, including 94 (56.6%) in CBT+ and 48 (57.8%) in DBT‐BED.

### 2.2 Interventions

#### 2.2.1 DBT‐BED

DBT‐BED teaches mindfulness, emotion regulation and distress tolerance skills, in order to replace binge eating as a way of coping with negative affect (Safer et al. 2009; Telch et al. 2001). The therapy consists of 20 weekly, two‐hour group sessions for a maximum of nine participants who start and end together (i.e., a closed group format). It includes weekly weighing and some attention is paid to a balanced eating pattern and regular physical exercise. One follow‐up session was conducted, 6 months post‐treatment.

#### 2.2.2 CBT+

CBT+ consists of 20 weekly treatment ‘days’, totaling 3 h and 45 min each, comprising weekly weighing and three modules: (1) Discuss self‐monitoring of eating behavior; (2) challenge thoughts and conduct behavioral experiments; (3) increase body‐awareness and promote regular exercise. A group is formed by a maximum of nine patients, with new patients starting every 10th week (i.e., a half open group format). Some attention is paid to emotion regulation. Also, six optional partner support meetings were included (90 min each). One follow‐up session was planned for all participants, 6 months after treatment. If evaluated as necessary on clinical grounds, five extra group sessions could be added to prevent relapse.

#### 2.2.3 Therapists

Therapists were trained and experienced in CBT+ as this is the treatment as usual (TAU) at the center. In the CBT‐program, each treatment‐cycle was led by a team of a psychiatric nurse (module 1), a psychologist (module 2) and a psychomotor therapist (module 3). Peer consultation was provided to ensure adherence to the program. For DBT‐BED, two trained psychologists/psychotherapists conducted each DBT treatment‐cycle. They were supervised monthly by a leading DBT‐expert. For more details, see Lammers et al. (2020).

### 2.3 Measures

Self‐report measures at baseline were used to assess main (predictor) and moderator effects.

#### 2.3.1 Overvaluation of Shape/Weight on Self‐Esteem

Consistent with earlier studies (Grilo et al. 2010; Messer and Linardon 2021), two specific items from the Eating Disorder Examination Questionnaire (EDE‐Q: Fairburn and Beglin 2008) were used to assess overvaluation of shape (i.e., ‘Over the past 4 weeks, has your shape influenced how you feel about (judge, think, evaluate) yourself as a person?’) and overvaluation of weight: (i.e., ‘Over the past 4 weeks has your weight influenced how you feel about (judge, think, evaluate) yourself as a person?’). Following Kenny and Carter (2018), we constructed a composite score of the two items by averaging the scores. Higher scores indicate greater severity. Internal consistency in the current sample was good (Cronbach's alpha = 0.928).

#### 2.3.2 Concern About Shape and Weight

Concerns about shape and weight were measured using the EDE‐Q subscales Shape Concern and Weight Concern. Because ‘overvaluation of shape and weight’ is a central concept in the CBT‐model (Fairburn et al. 2003) and considered distinct from shape and weight concerns (Grilo et al. 2013; Masheb et al. 2006; Wade et al. 2011) both scale scores were calculated without the respective overvaluation items. Subscale‐scores are calculated by summing the individual item‐scores divided by the number of subscale‐items. Higher scores indicate greater severity. The EDE‐Q has good psychometric properties (Aardoom et al. 2012; Berg et al. 2012). Internal consistency in the current sample was acceptable for shape concern and questionable for weight concern without the overvaluation items (Cronbach's alphas = 0.764 and 0.618 respectively).

#### 2.3.3 Dietary Restraint

The intention to limit food intake/dietary restraint was assessed with the 5‐item EDE‐Q subscale Restraint. Internal consistency in the current sample was adequate (*α* = 0.715).

#### 2.3.4 Self‐Esteem

The subscale Low Self‐Esteem (6 items) of the Eating Disorder Inventory (EDI‐3: Garner and Van Strien 2015) was used to assess self‐esteem. Subscale scores are calculated by summing the individual item‐scores. Higher scores indicate lower self‐esteem. The EDI‐3 has good psychometric properties and can be used in eating disorder patients (Clausen et al. 2011; Lehmann et al. 2013). In the current sample, the internal consistency was good (*α* = 0.876).

#### 2.3.5 Difficulties in Emotion Regulation

The 8‐item subscale Emotional Dysregulation of the EDI‐3 was used to measure the tendency toward poor impulse regulation and mood intolerance. Higher scores indicate more dysregulation in emotion. Internal consistency in the current sample was questionable (*α* = 0.651). The 7‐item subscale Difficulty Identifying Feelings of the Toronto Alexithymia Scale (TAS‐20: Bagby, Parker, and Taylor 1994; Bagby, Taylor, and Parker 1994) was used to assess difficulties in identifying feelings and distinguishing between emotional and physical sensations. Subscale scores are calculated by summing the individual item‐scores. Higher scores indicate more difficulties. Although the TAS‐20 has been subject to considerable debate as a measure of alexithymia, this specific subscale had reasonably good internal consistency (*α* = 0.792 in the current sample) and a satisfactory test–retest reliability in psychiatric outpatients (Kooiman et al. 2002).

#### 2.3.6 Negative Affect

The BDI‐II (21 items) assesses the severity of depressive symptoms over the last week with a higher total score indicating a higher level of depression. The reliability and validity of the BDI are good (Beck et al. 1996). Internal consistency in the current sample was good (*α* = 0.903). The SCL‐90 Anxiety subscale (10 items) assesses the severity of anxiety symptoms over the last week (Arrindell and Ettema 2003). Higher scores indicate higher levels of anxiety. Internal consistency in this sample was good (*α* = 0.873).

#### 2.3.7 Emotional Eating

The subscale Emotional Eating (13 items) of the Dutch Eating Behavior Questionnaire (DEBQ: Van Strien et al. 1986) was used to assess the desire to eat in response to negative emotions. Higher scores reflect higher levels of emotional eating. Internal consistency and factorial validity of the DEBQ are good (e.g., Barrada et al. 2016) and the reliability and validity are rated as good (enough) (COTAN 2013). Internal consistency in the current sample was good (*α* = 0.904).

#### 2.3.8 Outcome

Baseline, EOT, and six‐month FU reports of the number of OBE episodes on the EDE‐Q were used to assess the outcome: the reduction in OBE episodes from baseline.

### 2.4 Statistical Analysis/Analytic Approach

First, background, outcome, and predictor−/moderator variables were compared per condition with independent samples *T*‐tests and Chi‐square difference tests. Logistic regression was used to check whether participants who completed the follow‐up measurement differed from participants that dropped out on variables that were used in prediction−/moderation analyses. Generalized linear models based on a negative binomial distribution (for count data) with log link were used to evaluate predictors and moderators of treatment outcome for objective binge episodes at EOT (*n* = 203) and FU (*n* = 142). Separate models, each using list wise deletion, were run for each measurement timepoint (EOT and FU) in combination with each moderator. The models tested main effects for treatment (CBT or DBT), main effects for baseline variables (predictor effects), and interaction of treatment‐by‐baseline variables (moderator effects). By including the baseline covariate for objective binge episodes, we are analyzing residualized change in binge episodes from baseline to end‐of‐treatment and from baseline to follow‐up. This method is preferable to change scores because the residualized change score is uncorrelated with the baseline score (in contrast to calculated change score) (Cohen et al. 1983). Alpha correction for multiple comparisons was controlled using the false discovery rate (FDR; Benjamini and Hochberg 1995), with an overall FDR of 0.05. Effect size information for predictors and moderators is expressed as the *incidence rate ratio* (IRR), the increase in incidence rate for each one unit change in the predictor/moderator. IRR values of 1.22, 1.86, and 3.00 can be interpreted as small, medium, and large, respectively (Olivier et al. 2017). Graphs of significant interactions were created using regression coefficients to generate predicted scores across the observed range of the moderator. Data and study materials are available from the first author (ML).

## 3 Results

### 3.1 Descriptive Statistics

Demographic characteristics and baseline assessments of OBE frequency and moderators are provided in Table 1. Demographic variables did not differ between treatment groups except for education, with the CBT+ group obtaining a near significant (*p* = 0.058) higher percentage university‐level education (10.6% vs. 2.8%). In the DBT‐BED group, a significantly higher percentage (*p* = 0.007) lived with a partner/spouse (71.8% vs. 51.5%). Baseline OBE frequency did not significantly differ between participants in DBT‐BED or CBT+. Also, pretreatment levels of moderator variables did not differ between treatment groups (see Table 1). Logistic regression analysis showed that participants who completed the follow‐up measurement did not differ from participants that dropped out of the study on variables that were used in prediction−/moderation analyses.

**TABLE 1:** Baseline demographic and clinical characteristics.

Variable	CBT+ (*N* = 132)	DBT‐BED (*N* = 71)	*p*‐values (2‐sided)	Effect size	95% CI
Age (mean, SD)	35.24 (10.68)	37.63 (10.56)	ns	*d* = 0.22	−0.06, 0.51
Female (*n*, %)	117 (88.6)	66 (93.0)	ns	*Φ* = 0.07	−0.06, 0.20
Dutch nationality (*n*, %)	128 (97)	70 (98.6)	ns	*Φ* = 0.05	−0.07, 0.17
University education (*n*, %)	14 (10.6)	2 (2.8)	ns	*Φ* = −0.14	−0.28, 0.00
Living with partner/spouse (*n*, %)	68 (51.5)	51 (71.8)	0.007	*Φ* = 0.20	0.07, 0.33
BMI (mean, SD)	41.46 (7.00)	41.59 (6.46)	ns	*d* = 0.02	−0.27, 0.31
Full BED diagnosis (*n*, %)	112 (84.8)	66 (93.0)	ns	*Φ* = 0.12	−0.01, 0.25
Duration of illness (mean, SD)	15.95 (9.63)	17.21 (12.39)	ns	*d* = 0.12	−0.17, 0.41
OBE episodes (mean, SD)	7.98 (8.46)	6.56 (9.25)	ns	*d* = −0.16	−0.45, 0.13
EDI‐3 Bulimia (mean, SD)	15.12 (6.09)	15.03 (5.84)	ns	*d* = −0.02	−0.30, 0.27
EDE‐Q Global (mean, SD)	3.35 (1.01)	3.41 (0.99)	ns	*d* = 0.06	−0.23, 0.35
EDE‐Q Restraint (mean, SD)	1.56 (1.35)	1.71 (1.36)	ns	*d* = 0.11	−0.18, 0.40
Overvaluation of shape/weight (mean, SD)	4.94 (1.68)	5.09 (1.39)	ns	*d* = 0.09	−0.19, 0.38
EDE‐Q Weight Concern^a^ (mean, SD)	3.67 (1.24)	3.70 (1.27)	ns	*d* = 0.02	−0.26, 0.31
EDE‐Q Shape Concern^a^ (mean, SD)	4.22 (1.16)	4.25 (1.27)	ns	*d* = 0.03	−0.26, 0.31
DEBQ Emotional Eating (mean, SD)	3.91 (0.58)	4.00 (0.68)	ns	*d* = 0.15	−0.14, 0.43
SCL‐90 Anxiety (mean, SD)	18.17 (6.72)	18.71 (7.39)	ns	*d* = 0.08	−0.21, 0.37
BDI‐II Total (mean, SD)	23.08 (11.24)	22.85 (9.70)	ns	*d* = −0.02	−0.31, 0.27
EDI‐3 Low Self‐Esteem (mean, SD)	11.95 (5.60)	12.86 (5.18)	ns	*d* = 0.17	−0.12, 0.46
EDI‐3 Emotional Dysregulation (mean, SD)	5.83 (4.04)	6.08 (4.12)	ns	*d* = 0.06	−0.23, 0.35
TAS Difficulties Identifying Feelings	19.31 (5.39)	19.18 (5.38)	ns	*d* = −0.02	−0.31, 0.26

*Note*: CBT+ = intensive cognitive behavior therapy; DBT‐BED = dialectical behavior therapy for binge eating disorder; CI = confidence interval; BMI = body mass index; OBE = objective binge eating; EDI‐3 = Eating Disorder Inventory‐3rd edition; EDE‐Q = Eating Disorder Examination Questionnaire; Overvaluation of weight and shape, based on Kenny and Carter (2018): the average score of the EDE‐Q overvaluation of weight and shape items; DEBQ = Dutch Eating Behavior Questionnaire; SCL‐90 = Symptom Checklist‐90 items; BDI‐II = Beck Depression Inventory‐2nd edition; TAS = Toronto Alexithymia Scale.

^a^ The Eating Disorder Examination Questionnaire weight concern and shape concern scale scores were calculated without their respective overvaluation items. If the overvaluation items were to be included, the means of these scales would be as follows: CBT+ 3.93 (SD = 1.19), DBT‐BED 3.97 (1.21) (weight concern) and CBT+ 4.31 (SD = 1.16), DBT‐BED 4.36 (SD = 1.22) (shape concern). The difference between the two treatment groups is non‐significant for both weight concern and shape concern.

### 3.2 Generalized Linear Models: Decrease in OBE Episodes

**FIGURE 2:**
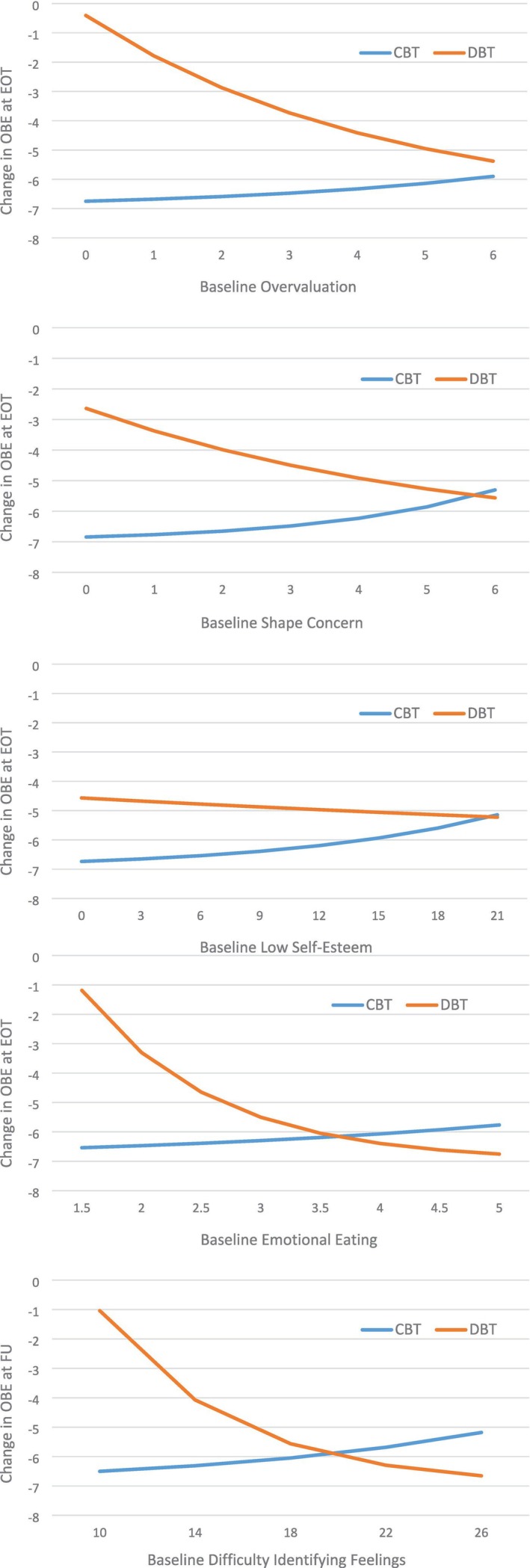
Graphs of significant moderators of treatment. *Source*: Graphs were created using regression coefficients to generate predicted scores across the observed range of the moderator.

Table 2 shows tests of prediction (i.e., main effect) and moderation (i.e., interaction) at end‐of‐treatment and follow‐up. Unadjusted significance values are presented in the table; significance after FDR adjustment are indicated with footnotes. Figure 2 shows graphs of significant interactions.

**TABLE 2:** Predictors and moderators of objective binge episode frequency at end of treatment and follow‐up.

Predictor/Moderator	Time point	Main effect						Interaction (X Treatment)					
		*b*	SE (b)	95% CI (b)	Sig	IRR	95% CI (IRR)	*b*	SE (b)	95% CI (b)	Sig	IRR	95% CI (IRR)
Overvaluation of Weight and Shape (EDE‐Q)	EOT	0.246	0.094	0.062, 0.431	0.009^a^	1.279	1.064, 1.539	−0.480	0.149	−0.771, −0.188	0.001^a^	0.619	0.462, 0.828
	FU	0.152	0.095	−0.034, 0.338	0.109	1.164	0.967, 1.402	0.074	0.210	−0.337, 0.486	0.723	1.077	0.714, 1.626
Dietary Restraint (EDE‐Q)	EOT	0.085	0.083	−0.077, 0.246	0.305	1.088	0.926, 1.280	−0.273	0.125	−0.517, −0.028	0.029	0.761	0.596, 0.972
	FU	−0.091	0.096	−0.280, 0.097	0.343	0.913	0.756, 1.102	−0.006	0.168	−0.336, 0.323	0.970	0.994	0.715, 1.381
Weight Concerns (EDE‐Q)	EOT	0.208	0.114	−0.015, 0.431	0.068	1.231	0.985, 1.539	−0.378	0.170	−0.711, −0.044	0.027	0.686	0.491, 0.957
	FU	−0.123	0.126	−0.370, 0.124	0.330	0.884	0.691, 1.132	0.231	0.233	−0.226, 0.688	0.322	1.260	0.798, 1.989
Shape Concerns (EDEQ)	EOT	0.396	0.137	0.129, 0.664	0.004^a^	1.487	1.137, 1.943	−0.581	0.167	−0.909, −0.253	0.001^a^	0.559	0.403, 0.776
	FU	−0.026	0.138	−0.296, 0.245	0.852	0.975	0.744, 1.277	−0.227	0.225	−0.669, 0.214	0.312	0.797	0.512, 1.238
Low Self‐Esteem (EDI‐3)	EOT	0.093	0.021	0.052, 0.135	< 0.001^a^	1.098	1.053, 1.145	−0.108	0.038	−0.181, −0.034	0.004^a^	0.898	0.834, 0.967
	FU	0.012	0.025	−0.037, 0.062	0.621	1.012	0.964, 1.063	0.044	0.042	−0.038, 0.125	0.292	1.045	0.963, 1.133
Emotion Dysregulation (EDI‐3)	EOT	−0.006	0.033	−0.071, 0.060	0.865	0.994	0.931, 1.062	0.001	0.053	−0.104, 1.05	0.992	1.001	0.902, 1.110
	FU	0.026	0.036	−0.045, 0.097	0.466	1.027	0.956, 1.102	−0.033	0.063	−0.156, 0.090	0.603	0.968	0.856, 1.095
Difficulty Identifying Feelings (TAS)	EOT	0.058	0.026	0.007, 0.108	0.025	1.059	1.007, 1.114	−0.048	0.041	−0.128, 0.031	0.231	0.953	0.880, 1.031
	FU	0.081	0.032	0.019, 0.143	0.011^a^	1.084	1.019, 1.154	−0.259	0.054	−0.365, −0.153	< 0.001^a^	0.772	0.694, 0.858
Depression (BDI‐II)	EOT	0.055	0.012	0.032, 0.079	< 0.001^a^	1.057	1.032, 1.082	−0.021	0.022	−0.064, 0.022	0.334	0.979	0.938, 1.022
	FU	0.037	0.013	0.010, 0.063	0.006^a^	1.037	1.011, 1.065	0.009	0.026	−0.041, 0.060	0.715	1.009	0.960, 1.062
Anxiety (SCL‐90)	EOT	0.045	0.021	0.005, 0.086	0.029	1.046	1.005, 1.090	0.044	0.032	−0.019, 0.107	0.167	1.045	0.982, 1.113
	FU	0.047	0.020	0.008, 0.086	0.018	1.048	1.008, 1.090	−0.070	0.034	−0.137, −0.003	0.040	0.932	0.872, 0.967
Emotional Eating (DEBQ)	EOT	0.281	0.202	−0.114, 0.676	0.163	1.325	0.892, 1.967	−1.185	0.390	−1.949, −0.421	0.002^a^	3.270	1.523, 7.019
	FU	0.846	0.308	0.242, 1.449	0.006^a^	2.330	1.274, 4.261	−0.754	0.434	−1.606, 0.097	0.082	0.470	0.201, 1.102

*Note*: b = unstandardized beta coefficient; SE = standard error; CI = confidence interval; sig = significance, *p*‐value; IRR = incidence rate ratio; EOT = end of treatment; FU = six‐month follow‐up; EDE‐Q = Eating Disorder Examination Questionnaire; EDI‐3 = Eating Disorder Inventory‐3rd edition; TAS = Toronto Alexithymia Scale; BDI = Beck Depression Inventory‐2nd edition; SCL‐90 = Symptom Checklist‐90 items; DEBQ = Dutch Eating Behavior Questionnaire; Overvaluation of weight and shape, based on Kenny and Carter (2018): the average score of the Eating Disorder Examination Questionnaire overvaluation of weight and shape items; Weight concerns/Shape concerns = the Eating Disorder Examination Questionnaire weight concern and shape concern subscale scores were calculated without their respective overvaluation items.

^a^ Predictor/Moderator significant after false discovery rate alpha correction.

#### 3.2.1 End‐of‐Treatment

##### 3.2.1.1 Predictors

Smaller reductions in OBE frequency at end of treatment were significantly predicted by higher overvaluation of weight and shape, higher shape concerns, greater low self‐esteem and higher depressive symptoms. Other predictors were non‐significant.

##### 3.2.1.2 Moderators

Four significant moderators were found at EOT: shape/weight overvaluation, shape concerns, low self‐esteem and emotional eating. CBT+ seems to perform only slightly better for those with low values on these variables compared to those at the high end. However, in DBT‐BED, individuals with high scores on overvaluation, shape concern, low self‐esteem and emotional eating showed greater reductions in OBE frequency than those with low scores. Additionally, individuals with the highest emotional eating scores showed more decrease in OBE episodes with DBT‐BED than with CBT+. Emotional eating showed a large interaction effect at EOT, while all other effects were (very) small. No significant results were found at EOT for dietary restraint, weight concerns, emotion dysregulation, difficulty identifying feelings, depressive symptoms and anxiety.

#### 3.2.2 Six‐Month Follow‐Up

##### 3.2.2.1 Predictors

Smaller reductions in OBE frequency at six‐month follow‐up were predicted by greater difficulty in identifying feelings, higher depressive symptoms, and higher emotional eating (medium effect) at baseline. Other predictors were non‐significant.

##### 3.2.2.2 Moderators

Only difficulty in identifying feelings appeared to be a significant moderator of treatment at six‐month follow‐up. CBT+ seems to perform, only slightly, better for those with low values, whereas in DBT‐BED, individuals with high scores showed greater reductions in OBE frequency than those with low scores. Those with the most difficulties in identifying feelings had greater reductions with DBT‐BED than with CBT+ Figure 2 Table 2

## 4 Discussion

The original comparisons of DBT‐BED and CBT+ regarding reductions in OBE frequency (Lammers et al. 2020, 2022) revealed few differences at EOT or six‐month FU. The present study aimed to broaden this work by identifying subgroups that might profit more from one treatment or the other and by examining general predictors, irrespective of treatment type. Predictor/moderator variables were selected based on the conceptual models underlying DBT‐BED and CBT.

Overall, our findings support the benefit of matching patients with specific symptom presentations on shape/weight overvaluation, shape concerns, low self‐esteem, difficulties in identifying feelings or emotional eating to DBT‐BED or CBT+. Findings rather consistently indicate that the association between a significant moderator and outcome is stronger for DBT‐BED than CBT+ and that, for low values of the moderator variable, DBT‐BED seems to result in less symptom reduction compared to CBT+. Notably, patients with the highest levels of emotional eating or difficulties identifying feelings showed better binge‐eating outcome in DBT‐BED (at respectively EOT and FU). The latter findings are consistent with our hypothesis that patients with distinct emotion regulation problems would demonstrate greater symptom improvements in DBT‐BED than in CBT+. However, levels of negative affect (depression and anxiety) and emotional dysregulation did not show a significant moderator effect. This may imply that specific aspects of ‘emotion regulation problems’ signal differential treatment outcome effects whereas other aspects do not. Although future studies need to replicate the cross patterns we found, our findings suggest that treatment‐specific factors (i.e., emotion regulation skills in DBT‐BED) may influence outcome for specific subgroups.

The hypothesis that patients with distinct problems related to dieting, shape/weight or self‐esteem, would do better in CBT compared to DBT‐BED, was not supported. In fact, outcome at EOT was better in CBT+ than in DBT‐BED at the *lower* end of shape/weight overvaluation, shape concerns and low‐self‐esteem. Interestingly, in a comparable study (Anderson et al. 2020), low shape/weight overvaluation signaled greater reductions in OBE frequency in an *emotion*‐focused treatment compared to CBT. These contradicting findings underline the importance to better understand how shape/weight overvaluation might influence symptom change in BED treatments. Future research should address this. Additionally, in line with other studies (Anderson et al. 2020; Grilo et al. 2019), the current sample comprises individuals with BED and rather high levels of shape/weight overvaluation (overall M = 4,99 SD = 1,58). This in itself is interesting as overvaluation is not included in the DSM‐5 criteria for BED and has been advocated to include as a severity‐specifier (Forrest et al. 2022; Grilo et al. 2013). Also contrary to the hypothesis that patients with distinct problems related to dieting, shape/weight or self‐esteem, would do better in CBT, levels of weight concerns and restraint did not moderate binge‐eating outcome. Notably, in line with previous studies (Anderson et al. 2020; Blood et al. 2020; Grilo et al. 2011), levels of dietary restraint are low (mean = 1.61, SD = 1.36). This adds to findings that question the importance of restraint in the maintenance of binge eating in BED and suggest that other factors may play a more critical role (Dakanalis et al. 2015).

All variables that moderated treatment outcome also showed significant predictor main effects: higher shape/weight overvaluation, shape concern and low self‐esteem predicted less reduction in binge frequency at EOT, whereas higher emotional eating and difficulties identifying feelings predicted worse outcome at FU. Findings were generally in line with previous BED studies regarding shape/weight overvaluation (Anderson et al. 2020; Grilo et al. 2012; Masheb and Grilo 2008), emotional eating (Ricca et al. 2010) and difficulties in identifying feelings (Fichter et al. 2008; Lammers et al. 2015). In line with Wilson et al. (2010), but unlike several other studies (Grilo et al. 2012; Peterson et al. 2000), low self‐esteem was predictive of outcome at EOT in the current sample, while Masheb and Grilo (2008) found self‐esteem to predict post‐treatment eating disorder psychopathology but not binge frequency. Also, we found greater shape concern predictive of worse outcome. This is in line with earlier studies, linking greater baseline shape/weight concerns (Hilbert et al. 2007) and greater weight concerns to poorer treatment outcome (Grilo, Thompson‐Brenner, et al. 2021). Finally, depression was the only variable that consistently predicted, but not moderated, outcome at both EOT and FU: higher levels predicted worse outcome. This adds to the mixed evidence regarding the predictive value of baseline depression‐scores in BED treatment. Interestingly, studies with seemingly lower mean levels of baseline BDI‐scores (15.54 – 18.65 – 20.7) (respectively Grilo, Gueorguieva, and Pittman 2021; Lammers et al. 2015; Dingemans et al. 2007) do not find a significant association, whereas studies with relatively higher mean levels of baseline BDI‐scores (23.08/22.85–26.5) (respectively this study and Dingemans et al. 2020), do find significant associations. Perhaps differences in results can be attributed to differences in the levels of depression in the sample at study.

Of note is that, in this study, emotional eating showed a medium and large predictor/moderator effect respectively, while all other statistically significant effects were (very) small. This may justify more attention to emotional eating as a variable of interest in BED predictor/moderator research. Also, in the current study, significant main and interaction effects were observed for shape concern but not weight concern. Although the latter finding could be attributed to the questionable internal consistency of the EDE‐Q subscale, in Grilo, Thompson‐Brenner, et al. (2021), significant findings showed weight concern, *not* shape concern, to predict and moderate outcomes in CBT and CBT‐gsh for BED. This may lead one to underline an earlier recommendation (Grilo, Thompson‐Brenner, et al. 2021) that shape and weight concerns should be considered separately, whereas the finding that most of the shape and weight concern items form one factor in factor analyses (Peterson et al. 2007; Wade et al. 2008) pleads for the opposite, namely, combine the subscales in analyses (Wade et al. 2011). Overall, the current study adds to previous findings that body image constructs may be of special importance in predicting who will benefit more, or less, from (a specific) BED treatment (Anderson et al. 2020; Grilo et al. 2012; Grilo, Thompson‐Brenner, et al. 2021; Lammers et al. 2015) and underlines the need to address body image in treatment.

The current study has several strengths. These include the selection of predictors/moderators, based on the conceptual models underlying the treatments of interest. Second, the use of a large sample size at baseline (CBT+ *N* = 132, DBT‐BED *N* = 71) and third, the fact that the sample consisted of individuals who were referred to help for their eating disorder through regular channels and got treatment in everyday clinical practice. This makes the data relevant for other regular clinical settings. Also, both EOT and FU outcome were assessed, thus including the possibility to investigate if significant findings are sustained 6 months after treatment and if nonsignificant findings at EOT may turn out to be significant at FU.

Beside the strengths, some limitations should be noted. An important limitation is the fact that CBT+ involves more contact hours compared to DBT‐BED. This limits the ability to conclude that patients with specific symptom presentations do better in one or the other treatment due to particular treatment characteristics (like addressing the overvaluation of shape and weight on self‐esteem in CBT+). A dose‐matched comparison would be necessary to eliminate the influence of dose as such. However, findings indicate that some patients do better in the more intensive form of *CBT*, and others do better in the less intensive *DBT‐BED*. This provides reason to further explore the interactions between specific subgroups, CBT and DBT‐BED.

Next, conceptual overlap between the two treatments may have occurred and therefore moderator effects were possibly compromised. This is a consequence of valuing generalizability and staying close to clinical practice: DBT‐BED incorporates lifestyle interventions and CBT+ includes some emotion regulation strategies. Also, the dataset used was comprised of two different samples, one that was (quasi‐) randomized and one that was not randomized (Lammers et al. 2020, 2022). Within the non‐randomized group, people were referred to one or the other treatment for various reasons. As we did not systematically record the reasons, we can only speculate as to whether or how this may have obscured some of the findings. However, at baseline, we did not find group differences on target variables in the current study, nor in the two separate samples (Lammers et al. 2020, 2022). Also, the internal consistency of both the EDE‐Q subscale weight concern and the EDI‐3 subscale emotional dysregulation was relatively low (Cronbach's *α* = 0.651 and 0.618 respectively). This means that we may not have exactly measured what we intended to measure. Therefore, future research should include more specific measures of emotional dysregulation (e.g., difficulties in emotion regulation scale (DERS; Gratz and Roemer 2002) and/or ecological momentary assessment, Engel et al. 2016) as well as consider to combine shape and weight subscales, as suggested by others (Peterson et al. 2007; Wade et al. 2008). Although this study constitutes of a large group at baseline and EOT, attrition at FU reduced the sample size, limiting the power to detect significant effects at FU.

## 5 In Conclusion

Findings suggest that BED treatment outcomes could be enhanced by matching individuals with certain symptom presentations to treatment overall, and to DBT‐BED or CBT+ specifically. DBT‐BED may be particularly effective in reducing OBE episodes in patients with high baseline levels of emotional eating (at EOT) or with high difficulties identifying feelings (at FU). Concurrently, relative to CBT+, DBT‐BED may not be particularly effective in patients with low levels of emotional eating, difficulties identifying feelings, shape/weight overvaluation, shape concerns and low self‐esteem. However, the lack of consistent moderator findings over time along with the limitations of this study suggests that more research is needed in order to provide clinicians a solid foundation on which to base effective decisions about what works for whom. In general, DBT‐BED seems to be a less viable alternative to CBT+ for those patients with less severe psychopathology, and a promising alternative for those patients with more severe psychopathology.

## Author Contributions

**Mirjam W. Lammers:** conceptualization; data curation; investigation; methodology; visualization; writing – original draft; writing – review and editing. **Maartje S. Vroling:** conceptualization; data curation; investigation; methodology; supervision; writing – review and editing. **Ross D. Crosby:** conceptualization; formal analysis; visualization; writing – original draft; writing – review and editing [of former version]. **Suzanne H. W. Mares:** formal analysis; visualization; writing – review and editing. **Giel J. M. Hutschemaekers:** Writing – review and editing. **Tatjana Van Strien:** conceptualization; supervision; writing – review and editing.

## Ethics Statement

The design of the (Lammers et al. 2020) study was approved on October 10, 2011 by the Institution of Mental Health Medical Ethics Committee (METiGG: 11.109; CMO Radboud UMC: 2013/226) and was registered retrospectively on August 28, 2013, https://onderzoekmetmensen.nl/nl/trial/25741.

## Consent

Data from the (Lammers et al. 2022) study were collected as part of routine clinical practice. Patients agreed that we use these data for scientific purposes by written informed consent.

## Conflicts of Interest

T. van Strien, receives royalties from the DEBQ, a questionnaire used in the present study, R.D. Crosby served as a paid Statistical Consultant, for Health Outcomes Solutions, Winter Park, Florida, USA.

## Data Availability

The data that support the findings of this study are available from the corresponding author upon reasonable request.
